# Dietary Manipulations Concurrent to Endurance Training

**DOI:** 10.3390/jfmk3030041

**Published:** 2018-07-25

**Authors:** Jeffrey Rothschild, Conrad P. Earnest

**Affiliations:** 1 TriFit Performance Center, Santa Monica, CA 90404, USA; 2 Exercise and Sport Nutrition Laboratory, Texas A&M University, College Station, TX 77843, USA

**Keywords:** endurance, training, adaptations, carbohydrate, LCHF

## Abstract

The role of an athlete’s dietary intake (both timing and food type) goes beyond simply providing fuel to support the body’s vital processes. Nutritional choices also have an impact on the metabolic adaptations to training. Over the past 20 years, research has suggested that strategically reducing carbohydrate (CHO) availability during an athlete’s training can modify the metabolic responses in lieu of simply maintaining a high CHO diet. Several methods have been explored to manipulate CHO availability and include: Low-carb, high-fat (LCHF) diets, performing two-a-day training without glycogen restoration between sessions, and a “sleep-low” approach entailing a glycogen-depleting session in the evening without consuming CHO until after a morning training session performed in an overnight fasted state. Each of these methods can confer beneficial metabolic adaptations for the endurance athlete including increases in mitochondrial enzyme activity, mitochondrial content, and rates of fat oxidation, yet data showing a direct performance benefit is still unclear.

## 1. Introduction

The endurance athlete’s diet can impact the metabolic adaptations to training by enhancing or blunting cellular responses to exercise-induced perturbations. A high carbohydrate (CHO) diet has been traditionally promoted for these athletes in order to maximize muscle and liver glycogen stores, along with the use of exogenous CHO fuels during both training and competition [1]. Over the last 20 years, data suggesting benefits from purposely and strategically reducing the availability of CHO during some or all of an athlete’s training sessions has appeared with increased frequency in the literature. Such manipulations can modify the metabolic responses to training and result in more favorable responses to exercise stimuli [2]. To date, several methods have been used to strategically manipulate CHO availability surrounding training including: A low-carb, high-fat diet (LCHF), two-a-day training without glycogen restoration between training sessions, and a “sleep-low” approach where the athlete performs a glycogen-depleting session in the evening without consuming CHO until after a morning training session performed in an overnight fasted state. Each of these methods can confer beneficial metabolic adaptations to the endurance athlete including increases in mitochondrial enzyme activity, mitochondrial content, and rates of fat oxidation, though data showing direct performance benefits are less clear. 

To optimize training adaptations, the interplay of training volume, training intensity, and dietary CHO must be considered. For example, regulation of peroxisome proliferator-activated receptor c coactivator-1α (PGC-1α) protein content appears to be primarily dependent upon training intensity and is not affected by CHO availability [3,4,5,6]. Changes in citrate synthase (CS) activity are primarily affected by training volume [7] and largely unaffected by CHO intake [8], though some studies have shown an augmented training response with dietary manipulation [9,10]. A number of studies have shown that modulating CHO availability can enhance the training-induced responses of key signaling proteins involved in mitochondrial biogenesis such as p38 mitogen-activated protein kinase (MAPK) [3], 5′ AMP-activated protein kinase (AMPK) [11], and p53 [4]. Therefore, the objective of this review is to examine the impact of dietary manipulation on the adaptations to endurance training.

## 2. Low-Carb, High-Fat

A LCHF dietary approach has been explored by endurance athletes due to its ability to increase rates of fat oxidation and reduce reliance on CHO during prolonged exercise [12]. Fat metabolism during exercise is a complex process involving many sites of regulation that may be affected by dietary intake including the release of free fatty acids (FFA) from adipose tissue and delivery to working muscle, transport of fat into the cell, binding and transport of fat in the cytoplasm, transport of fat into the mitochondria, and the regulation of intramuscular triglyceride (IMTG) synthesis and breakdown [13,14]. A LCHF diet increases the release of FFA from adipose tissue at rest and during exercise [15], which can be transported to the muscles and oxidized or re-esterified and stored into the IMTG droplets located next to the mitochondria. Adipose derived FFA increase rapidly during low to moderate intensity exercise with a greater proportion of FFA undergoing beta oxidation rather than re-esterification [16], while other fat sources, such as IMTG, are stimulated as exercise intensities increase [17]. A LCHF diet can increase resting IMTG levels by 50–123%, independent of glycogen availability [13,18,19,20], with just two days of LCHF being sufficient to increase levels by 36% in trained participants [21]. Training status appears to affect the extent of these adaptations, as untrained males on LCHF (55–60% fat) for 16 d showed no changes in IMTG levels [22]. 

When fuel availability is altered, intracellular metabolites regulating key cellular enzymes will also be altered. For example, five days of LCHF resulted in 12–17% increases in fatty acyl translocase (FAT/CD36) protein content, indicating an increased capacity for sarcolemmal and/or mitochondrial membrane fatty acid uptake [23,24]. In contrast, carnitine palmitoyltransferase-1 (CPT1) was not changed after 5 or 6 d of LCHF [23,24,25] but increased after 10 and 15 d [26]. The activity of β-hydroxyacyl-CoA dehydrogenase (β-HAD), which plays a key role in beta oxidation, was unchanged after 6, 15, and 28 d of LCHF [19,25,26] but increased by 120% after 7 weeks of LCHF [8]. Gene expression of *β-HAD* increased after 5 d of LCHF, suggesting a longer duration may be needed to see increased functional protein concentrations [24]. No changes have been observed in CS activity with LCHF [8,19,23,25,26], which is increased by training but largely unaffected by diet [8]. 

A LCHF dietary approach can dramatically increase rates of maximal fat oxidation, from ~0.59 g per minute on a mixed diet [27] to ~1.5 g per minute on LCHF with some athletes exceeding 1.9 g per minute after a three week intervention [28,29]. Some disagreement in the literature exists concerning the source of fat for the increased fat oxidation observed with LCHF diets. A number of studies have found that plasma VLDL-TG in combination with increased FFA uptake accounts for much of the increased fat oxidation observed during exercise after LCHF diet adaptation [30,31,32], while other research has shown the increases are the result of elevated IMTG concentration and not increased adipose tissue lipolysis or plasma FFA [21]. Reasons for divergent findings may be the length of adaptations to LCHF, CHO content of the meal consumed prior to the exercise testing, and fitness level, as trained endurance athletes have a substantially increased capacity to store and utilize IMTG during exercise [33]. Along with increased rates of fat oxidation on LCHF diets, decreased rates of CHO oxidation are observed due to reductions in muscle glycogen storage and breakdown, decreased activity of pyruvate dehydrogenase (PDH), reduced plasma glucose concentrations, and reduced intake of exogenous CHO during exercise [24,25,26,28,29,34,35,36,37,38].

The majority of research has shown that LCHF reduces levels of stored muscle glycogen and thus its contribution during exercise [34,37,39,40]. However, an observational study of ultra-endurance athletes on a long-term (>9 month) LCHF diet found no differences in muscle glycogen storage or utilization while running at 64% VO_2max_ for 180 min [28]. The authors speculated this could be attributed to hepatic gluconeogenesis during exercise and that lactate and/or glycerol could have provided a source of carbons for glycogen synthesis during recovery as levels of both were higher at the end of exercise and decreased during recovery. Another long term (>8 months) LCHF study observed decreased breakdown of hepatic glycogen during exercise, though hepatic gluconeogenesis did not increase compared with high CHO athletes [37]. However, the gluconeogenic precursors were likely to be more dependent on glycerol in the LCHF group and lactate in the high CHO group. Using a combination of muscle biopsy and CHO tracers, there was a 4-fold reduction in muscle glycogen use and 3-fold reduction in blood glucose oxidation during moderate intensity cycling after 4-week LCHF, with glycerol from triglycerides providing up to 40% of the glucose consumed and gluconeogenesis from lactate, pyruvate, and glucogenic amino acids providing the additional substrate needed to maintain plasma glucose levels and allow glycogen restoration [34].

The effects of LCHF on performance have been equivocal, with some studies showing benefit [38,41,42] and the majority showing no changes [20,35,36,38,43,44] or decrements [15,44,45,46,47]. Fourteen days of LCHF was able to attenuate the decline in power during a 100 km time trial that was observed with high-CHO, though overall performance times were not significantly different [41]. However, performance decrements have been observed during shorter, higher-intensity efforts. These include 6% lower power output on a Wingate test [47], 15% less distance run during the yo-yo intermittent recovery test [47], and trends toward significantly lower 1 km time trial performance (*p* = 0.07) [44] and 15-min time trial (*p* = 0.11) performance [41]. Further research is needed on the individual responses to LCHF, as several studies that showed no mean differences have had participants with favorable performance improvements [34,38,45].

To counteract the negative effects on performance observed with reduced glycogen levels, a model of dietary periodization was established that included a 5–14 day LCHF fat adaptation phase followed by a 1–3 day CHO restoration phase [18,35,36,41,42,43,48]. The concept was that the CHO restoration phase would allow glycogen to be replenished while retaining the skeletal muscle responses to fat adaptation [35]. Indeed, multiple studies have shown elevated values of fat oxidation compared with baseline, though the levels are less than what is observed prior to CHO restoration [18,35]. Ten days of LCHF followed by three days of CHO-loading improved 20 km time trial performance by 4.3% following cycling for 2.5 h at 70% VO_2max_ [42], though similar studies have not shown performance improvements [35,36,43,44]. LCHF diets with CHO restoration reduce rates of muscle glycogen utilization, even after replenishing glycogen stores [18,35,42], which can be explained by a reduction in PDH after five days of LCHF that remained after one day of CHO restoration [48]. These adaptations to LCHF can persist through at least 36 h of dietary strategies to restore muscle and liver glycogen and increase CHO availability during exercise, though the exact time course of this period is currently unknown [49]. 

Well-trained athletes are able to oxidize more fat for fuel during exercise compared with recreationally trained or untrained individuals [50,51], and correlations have been observed between maximal rate of fat oxidation and endurance performance [52]. At the same time, it is clear that LCHF dietary interventions are able to drastically increase rates of fat oxidation regardless of training status [28,29,53]. Arguments in favor of trying to increase fat-burning capacity focus around the ability to utilize the large stores of endogenous lipids found even in very lean athletes, while preserving the relatively limited supply of muscle and liver glycogen. Yet despite this theoretical advantage, measurable performance improvements from deliberately increasing fat burning capacity have been elusive. One reason may be the decreased oxygen efficiency with the oxidation of fat compared with CHO. It has long been known that CHO yields more ATP per liter of oxygen compared with fat [54,55], resulting in an increasing oxygen consumption for a given running pace/cycling power output as the respiratory exchange ratio shifts downward. A 3-week study in elite race-walkers following LCHF found that increased rates of fat oxidation were accompanied (and offset) by an increased oxygen cost of walking, resulting in a lack of improvement in a 10 km race while performance improvements were observed in high-CHO and periodized-CHO groups [29]. Similar findings of decreased economy have also been observed in recreational runners on LCHF [53], while three days of high-CHO diet improved cycling gross efficiency compared with low and moderate-CHO diets [56]. 

Throughout the literature, there is a challenge in determining whether the adaptations to LCHF are driven by high fat intake or reduced CHO availability. To separate the effects of low CHO from high fat on mitochondrial respiration, well-trained cyclists consumed 5 days of isoenergetic high fat or high-protein diets (~67% of daily energy intake, with CHO clamped at <20%) [23]. The high-fat diet was able to increase rates of whole-body fat oxidation to a greater degree than the high-protein diet. In addition, the high-fat diet decreased skeletal muscle mitochondrial respiration driven by both FFA and pyruvate, as well as uncoupled respiration during exercise compared with high-protein. One day of high CHO intake was sufficient to return mitochondrial respiration levels to baseline, while no changes in mitochondrial respiration were observed after 5 days of the high-protein diet. Rates of substrate oxidation in both groups also returned towards baseline after one day of high CHO. This study suggests that high dietary fat intake, rather than low-CHO intake, is the primary driver in the reductions in mitochondrial respiration and increases in whole-body rates of fat oxidation on a LCHF diet. These results are in line with research in untrained males where LCHF blunted the exercise-induced increase in uncoupled respiratory capacity rates [22]. 

Overall, LCHF diets can increase the amount of fat utilized during exercise, though performance at higher exercise intensities may be compromised (Table 1). Due to the complex nature of metabolic regulation it is still unknown what the exact limiters of fat oxidation in skeletal muscle during increased exercise intensities are. Future research should continue looking at mechanisms of action for the diet-induced differences in enzymes that regulate mitochondrial substrate flux as well as the time course of CHO washout after LCHF + CHO interventions. 

## 3. CHO Manipulation

Traditional approaches to endurance training diets have promoted high CHO availability before, during and after training sessions to allow the athlete to train longer and harder in order to maximize the adaptive response. However, research over the past 10–15 years has demonstrated an increased role for CHO both as a fuel source and in metabolic signaling [57,58]. Deliberately restricting CHO before, during, and/or after exercise can result in positive training adaptations beyond what would be seen with high-CHO including increased mitochondrial enzyme activity, mitochondrial content, and rates of fat oxidation, with some research showing improvements in exercise capacity (Table 1). It has become apparent that the nutritional strategies for optimal performance (e.g., maximizing CHO availability) may not be the same as the strategies for maximal training adaptations (e.g., strategic CHO reduction). For example, exercise undertaken in a glycogen-depleted state increases the phosphorylation of AMPK in the post-exercise window, which directly phosphorylates PGC-1α, a protein that can induce mitochondrial biogenesis, angiogenesis, and increases in fat oxidation [4,11,59,60,61]. This has led to the concept of ‘train low, compete high’ which features training sessions performed under conditions of reduced CHO availability while CHO reserves are restored prior to and during competition. 

### 3.1. Twice Daily Training

The idea that training with reduced CHO availability can beneficially impact training adaptations came from observations that an acute bout of exercise undertaken with reduced muscle glycogen led to enhanced expression of genes related to substrate utilization and mitochondrial biogenesis [5,62]. This was further explored using a single leg training protocol, with twice-daily training and CHO restriction between sessions every other day demonstrating greater increases in CS maximal activity, exercise time to fatigue, and resting muscle glycogen content compared with once daily training with high CHO availability [9].

Several three-week studies in trained cyclists looked at twice daily training with CHO intake restricted between sessions compared with participants training once daily with high CHO availability [63,64]. Although performance improvements were similar between groups, the twice daily training groups showed greater increases in CS maximal activity, β-HAD, cytochrome oxidase subunit IV (COX-IV), and whole body fat oxidation during steady state cycling which was due to increased oxidation of IMTG rather than plasma FFA [63,64]. Increases in the activity of mitochondrial enzyme succinate dehydrogenase (SDH) and reductions in glycogen utilization have also been observed during a similar 6-week protocol, though performance improvements were not affected by diet [65]. In contrast, a two-week study in healthy but untrained participants comparing twice daily training while either ingesting high-CHO (195 g) or low-CHO (17 g) during the three-hour period between workouts found that post-intervention time trial performance was greater in the group training with low-CHO though there were no differences between groups in mitochondrial enzymes CS or COX-IV [66]. Possible reasons for these contrasting results include differences in training status and training intensity. The two studies using well-trained cyclists showed no performance benefits from low-CHO training [63,64], while a study using untrained participants did find favorable improvements [66]. Total work completed during training sessions may also impact study outcomes. When participants were instructed to self-select training intensity (e.g., 8 × 5 min at maximum self-selected effort), power output was 8% lower with low-CHO training and no performance changes were seen [63,64]. When training intensities were prescribed to ensure both groups had trained at the same power output, additional performance improvements were seen with low-CHO training [66]. Also, though not directly measured in these studies performance improvements may have resulted from changes in mitochondrial respiratory function, which are not always associated with training-induced changes in mitochondrial content [67]. Finally, it is noteworthy for athletes and coaches that the declines in exercise capacity observed while training in a CHO depleted state [64] can be recovered in part by caffeine [68,69], CHO mouth rinse [70], or the combination of both [71].

### 3.2. Sleep Low

Although the training adaptations with reduced compared with high-CHO availability have shown favorable adaptations at the molecular and cellular levels, reliable improvements in real-world performance have been elusive. This may be because dietary strategies that reduce CHO availability also reduce the ability of the athletes to effectively complete high-intensity interval training (HIIT) sessions [64]. Because of this, and in light of favorable adaptations observed with post-workout CHO restriction [3], a “sleep-low” protocol was established. During this protocol, athletes consume a high-CHO diet prior to an evening session of HIIT, go to sleep without consuming any post-workout CHO and then complete a low-intensity workout the next morning in a fasted state to optimize fat-burning adaptations [72]. Compared with a CHO-fed control group, the sleep-low group increased fat oxidation during 2 h of steady state cycling along with elevated CPT-1 and greater increases in resting phosphorylation of AMPK and p38 MAPK, markers of mitochondrial adaptation [72]. In two follow-up studies lasting one and three weeks, respectively, participants completed a sleep-low protocol three days per week [73,74,75]. After three weeks the sleep-low group had improvements in submaximal cycling efficiency and supra-maximal cycling to exhaustion along with a 3% improvement in 10 km running performance and decreased fat mass, while no performance changes were seen in the control group [73]. The one week study saw a similar (~3%) improvement in 20 km cycling time trial performance [75]. It was also found that the 3-week protocol had minimal effects on markers of sleep quality and immune function [74].

While the initial sleep-low study had participants completely refrain from eating after the evening HIIT session [72], a key difference in follow-up studies [73,74,75] was the provision of a protein-containing, non-CHO meal after the evening HIIT. However, concern regarding the long-term impact of post-workout CHO restriction is warranted as levels of p70S6K (a key regulator of the skeletal muscle response to exercise) are suppressed following exercise and restored with intake of CHO but not from ingestion of protein-only [76]. Post-exercise fat and protein consumption also reduced p70S6K activity, in contrast with increased activity observed with high CHO [77]. This, along with elevated rates of post-exercise myofibrillar muscle protein synthesis and p70S6K phosphorylation with CHO and protein [78] suggests that athletes who withhold CHO prior to and/or during training sessions should consume both CHO and protein post-workout in order to maximize the skeletal muscle adaptive response. Thus, more research is needed to reconcile the observed short-term performance benefits from sleep-low protocols with any potential negative impact on the post-exercise muscle adaptive responses. This apparent contradiction may be explained by differences in the skeletal muscle response to acute vs. chronic HIIT [79], and the findings of a meta-analysis that total daily protein intake, rather than timing, was the strongest predictor of exercise-induced muscle hypertrophy [80]. For an in-depth review of molecular adaptations to exercise the reader can be directed to several recent reviews [81,82,83].

### 3.3. Fasted Training 

Performing exercise after an overnight fast is another way to alter CHO availability and augment the adaptive response to endurance training. In contrast with twice daily training sessions performed without CHO restoration that result in depleted muscle glycogen levels for the second bout of exercise, overnight fasting reduces liver, but not reduced muscle glycogen, concentrations [84]. Blood glucose concentration may be maintained at normal levels during exercise after an overnight fast despite the depletion of liver glycogen, likely due to increased gluconeogenesis and decreased utilization of glucose in muscle as a result of lowered PDH activity [85]. 

A recent review and meta-analysis looking at the effects of fed vs. fasted aerobic training on substrate usage found that fasted exercise induces higher fat oxidation than exercise performed in the fed state, though no differences in plasma FFA were found [86]. However, muscle glycogen levels need to be determined when drawing conclusions on substrate oxidation between fasted and non-fasted training as no differences were observed in fat oxidation during the first 90–120 min of fasted compared with non-fasted exercise when muscle glycogen levels begin at the same level [87,88], despite reductions in lipolysis and plasma FFA concentration [88] as well as elevated insulin levels from CHO ingestion during moderate-intensity exercise [89]. Steady state cycling in a fasted state can also cause the breakdown of IMTG in type I muscle fibers, which was completely blunted in a CHO-fed state [90]. Broad conclusions from the available research are also challenged by variations among testing protocols. For example, IMTG usage was increased after 6 weeks of fasted (but not fed) training when tested in the fasted state [10] while another 6-week study resulted in no differences in fat oxidation rates or IMTG breakdown during exercise in a fed state while also providing additional CHO [91], suggesting a lack of carryover to real-world application. Mechanisms for increased fat utilization during fasted exercise can include changes in adipose tissue mRNA expression of *PDK4*, adipose triglyceride lipase, *HSL*, *β-HAD*, *CPT-1*, *FAT/CD36*, *GLUT4,* and insulin receptor substrate 2, all of which were lower in response to fed compared with fasted exercise [91,92].

A review and meta-analysis looking at the effects of fed vs. fasted exercise on performance and post-exercise metabolism found that exercise in the fed state enhanced prolonged, but not shorter duration aerobic exercise performance, while fasted exercise increased post-exercise circulating FFA compared to fed exercise and pre-exercise feeding blunted signaling related to mitochondrial adaptation such as β-HAD and CS [93]. Performance changes after 6-week endurance-training programs were not different between participants completing the training in the fed or fasted states [10,91], though only fasted training led to increases in maximal activity of CS and β-HAD while also preventing an exercise-induced drop in blood glucose [10]. In contrast, a 6-week study in overweight women performing HIIT in the fed or fasted state found no effect of fasting vs. fed state on training-induced increases in CS, β-HAD, or GLUT4 protein content [94]. It is possible that a different result would have been observed from higher-volume, lower-intensity training as CS activity is increased by volume and not intensity [7]. Sex differences may be seen with fasted training, as a 4 week study in untrained males and females found training-induced changes in CS and β-HAD were not different between fed and fasted training but women had greater increases with fed training while men had greater increases with fasted training [95]. That study also found the fasting group had greater training-induced increase in VO_2max_ and resting muscle glycogen levels [95]. The majority of studies using sedentary and/or overweight/obese populations have found no changes in post-exercise glucose, insulin, or FFAs between fasted and fed conditions, further highlighting the potential role of training status on metabolic flexibility [93,96].

### 3.4. Periodized Carbohydrate

Perhaps the most pragmatic approach may be to use a periodized CHO intake, which refers to performing some training sessions with high CHO availability (high muscle glycogen, CHO feeding during session) and others with low CHO availability (low pre-exercise glycogen, avoidance of intra-workout CHO, overnight fasted, or delayed post-session refueling). This allows high-intensity workouts to be completed at the highest possible work output and low-intensity training to be performed without CHO in order to maximize mitochondrial adaptations. CHO-restricted training sessions may be critical for maximizing fat oxidation, as ingestion of CHO during exercise decreases the expression of genes involved in lipid metabolism (e.g., *GLUT-4*, *PDK4*, *AMPK*, *CD36*, *CPT-1*, and *UCP3*) rather than increasing genes involved in carbohydrate metabolism [97], as well as blunting the interleukin-6 response to exercise [98]. A study in elite endurance athletes that compared LCHF to both high-CHO and a high but periodized CHO intake found that LCHF and periodized-CHO had greater losses in body mass during the 3-week intervention, while the performance improvements and measures of oxygen consumption in high-CHO and periodized CHO groups were similar and both more favorable than LCHF [29]. Another 4-week study that compared high CHO to a periodized CHO intake found no significant differences in the training effects between dietary approaches [99]. While a periodized approach to CHO intake seems to intuitively make sense, longer-term studies are needed to determine if there are favorable performance benefits compared with high-CHO. 

In summary, deliberately restricting CHO before, during, or after endurance training can lead to greater training stimuli at the cellular and molecular levels but poses additional challenges for the athlete who wishes to maintain their desired training intensities. On a practical level it is critical for athletes practicing “train-low” approaches to also include workouts that are fully fueled to be sure they retain their capacity to absorb and oxidize CHO without gastrointestinal distress [100,101], and maintain activity of the PDH enzyme complex [48]. Consuming protein prior to exercise with low-CHO availability may be a sensible strategy as consumption of protein before and during exercise does not impair FFA availability or whole body fat oxidation despite elevated insulin levels [102], and exercising with reduced muscle glycogen increases both muscle protein breakdown and the contribution of protein to energy production [103,104]. Effects of training in the fasted state are inconsistent, likely due to differences in training status, sex, and testing conditions.

Future work should attempt to further elucidate differences between CHO restriction surrounding low intensity and high intensity workouts and determine the effects of changing the order of low and high intensity workouts during twice daily training. Further examination into the role of training status on the effects of CHO restricted training as well as the time course needed to see real world performance effects would also be valuable for coaches and athletes. In addition, comparisons of the metabolic and performance responses of fasted (with high and low muscle glycogen) to LCHF interventions and protein-only feedings prior to exercise are warranted.

## 4. Personalization, Preference and Perception

Though most athletes acknowledge the importance of diet in optimal sports performance [105], the make-up of an optimal diet may be less clear. In an observational study of athletes across 13 sports that were seeking nutritional advice from on-site dietitians at a major international competition, CHO intake (based on 24-h recall) ranged from 1.0–9.0 g/kg per day, with a median value of 3.8 g/kg [106]. Studies in elite and sub-elite male Australian football and soccer players scored an average of 57–62% in a questionnaire designed to test their nutrition knowledge, while intake of CHO was lower than recommended and nutrition knowledge was positively correlated with fat-free soft tissue mass [107,108]. In a survey of competitive tennis players, only ~50% of players reported opting for CHO dominant meals prior to a match, while 39% of players reported consuming “nothing specific” on the day after a match [109]. This suggests a large knowledge gap between sports nutrition research-based recommendations and application among athletes and practitioners in the field.

Other research in elite endurance athletes has reported practices that more closely align with recommendations for athletes training at a high volume [110]. Professional male cyclists being observed during three consecutive days of high-intensity training consumed an average of 9.8–12.2 g/kg CHO per day [111], while elite female cyclists observed during a nine day training block reported an intake of 7.5–10 g/kg per day [112]. Another study observing professional cyclists during preseason training camp reported a slightly lower self-selected CHO intake of 6.7 g/kg per day [113]. A case study of elite marathon runners reported training sessions performed with low-CHO availability 1–3 times per week; 90% of those sessions being after an overnight fast and 10% being reduced glycogen training from twice daily training without CHO restoration [114].

A survey looking at the dietary practices of 48 elite male and female middle- and long-distance runners/race-walkers found that while nearly all athletes (96%) focus on adequate intake around key training sessions, only 26% report ever training in an overnight-fasted state, 11% report periodically restricting CHO intake, and only 30% ingest CHO during training sessions [115]. When dietary intake during one week of high intensity training camp was analyzed for periodization between hard and easy training days, females had greater energy intake on hard training days (204 vs. 187 kJ/kg/day) while males did not significantly change intake, and females showed a pattern of periodization of post-exercise CHO for key vs. easy training sessions (0.9 vs. 0.5 g/kg, respectively) while males had only modest periodization (1.3 vs. 1.0 g/kg, respectively) [116]. Furthermore, only 73% of athletes who reported in the survey that they focused on adequate fueling before key workouts were observed to meet the recommended target intakes, while post-workout CHO and protein targets were only met by 56% and 26%, respectively, of the 87% of athletes that reported paying attention to nutrition recovery after key workout sessions [116]. These findings suggest a potential lack of awareness surrounding the benefits of strategic CHO manipulation as well as incomplete execution of the latest guidelines encouraging a periodized nutrition approach to support optimal performance, recovery, and training adaptation [110]. Future research should look at not just the beliefs and practices of athletes, but of coaches and nutritionists to determine if current, evidence-based recommendations are being provided to athletes.

## 5. Conclusions

Dietary intake (both the timing and types of food) can impact the metabolic adaptations to training, though specific training responses can vary based on training status, pre-exercise glycogen levels, exercise intensity and duration, and post-workout feeding. Though direct performance benefits are challenging to measure, accumulating data suggests that partaking in some training sessions with low, and some training sessions with high, CHO availability may lead to optimal training outcomes. This can be accomplished by following a short-term low-carb, high-fat (LCHF) diet, performing twice daily training without glycogen restoration between sessions, a “sleep-low” approach where the athlete does a glycogen-depleting session in the evening and does not consume CHO until the following day, and undertaking AM training sessions in an overnight fasted state. 

## Figures and Tables

**Table 1:** Summary of adaptations to concurrent diet and endurance training strategies. Abbreviations and symbols: FAT/CD36: fatty acyl translocase; CPT-1: carnitine palmitoyltransferase-1; β-HAD: β-hydroxyacyl-CoA dehydrogenase; ↑: increased; ↓: decreased, Ø: nonsignificant change.

Dietary Intervention	Fat Oxidation	Carb Oxidation/ Glycogen Utilization	Glycogen Storage	CPT-1/FAT/CD36	β-HAD	Citrate Synthase	Performance
**LCHF**	↑	↓	Ø ↓	Ø ↑	Ø ↑	Ø	↑ Ø ↓
**Twice daily training**	↑	↓	↑	↑	↑	Ø ↑	Ø ↑
**Sleep Low**	Ø ↑	Ø ↓		↑			↑
**Fasted Training**	Ø ↑	Ø ↓	↑	↑	Ø ↑	Ø ↑	Ø ↓
